# Anti-correlation of HER2 and focal adhesion complexes in the plasma membrane

**DOI:** 10.1371/journal.pone.0234430

**Published:** 2020-06-08

**Authors:** Florian Weinberg, Mitchell Kim Liong Han, Indra Navina Dahmke, Aránzazu Del Campo, Niels de Jonge

**Affiliations:** 1 INM – Leibniz Institute for New Materials, Saarbrücken, Germany; 2 Department of Chemistry, University of Saarland, Saarbrücken, Germany; 3 Department of Physics, University of Saarland, Saarbrücken, Germany; Oregon State University, UNITED STATES

## Abstract

Excess presence of the human epidermal growth factor receptor 2 (HER2) as well as of the focal adhesion protein complexes are associated with increased proliferation, migratory, and invasive behavior of cancer cells. A cross-regulation between HER2 and integrin signaling pathways has been found, but the exact mechanism remains elusive. Here, we investigated whether HER2 colocalizes with focal adhesion complexes on breast cancer cells overexpressing HER2. For this purpose, vinculin or talin green fluorescent protein (GFP) fusion proteins, both key constituents of focal adhesions, were expressed in breast cancer cells. HER2 was either extracellularly or intracellularly labeled with fluorescent quantum dots nanoparticles (QDs). The cell-substrate interface was analyzed at the location of the focal adhesions by means of total internal reflection fluorescent microscopy or correlative fluorescence- and scanning transmission electron microscopy. Expression of HER2 at the cell-substrate interface was only observed upon intracellular labeling, and was heterogeneous with both HER2-enriched and -low regions. In contrast to an expected enrichment of HER2 at focal adhesions, an anti-correlated expression pattern was observed for talin and HER2. Our findings suggest a spatial anti-correlation between HER2 and focal adhesion complexes for adherent cells.

## Introduction

HER2, also named ErbB2, belongs to the epidermal growth factor receptor (EGFR) family, and is overexpressed in 20–30% of breast cancer cases [1]. High HER2 expression promotes proliferation, migration, and invasiveness of cancer cells, whereby HER2 is preferentially found at the plasma membranes in so-called ruffles [2–4]. These cellular processes are enhanced by the adhesion of tumor cells to their surrounding environment, the extracellular matrix (ECM). Adhesion molecules, such as integrins, form a signaling-competent focal adhesion complex once bound to the ECM [5]. It has been established that integrin- and HER2-signaling are functionally linked by sharing common signaling pathways that actively modulate cellular responses involved in cancer cell invasion [6–10]. It was found, for instance, that focal adhesion kinase (FAK) signaling was increased by overexpressing HER2 in cells with low endogenous HER2 [11]. Similarly, overexpression of focal adhesion proteins involved in cell-substrate adhesion, such as integrins or Col2a1, increased HER2 signaling and resistance to anti-HER2 therapy [12–14]. Vice versa, knockdown of integrins or inhibition of FAK signaling improved anti-HER2 therapy effectiveness [15–17]. It has been suggested by biochemical-, and microscopic methods that both proteins HER2 and integrin coexist in membrane clusters for non-adherent cells [18–20]. This co-localization was found to get lost under experimental conditions promoting the formation of focal adhesion [18]. On the contrary, HER2 and integrin were found to colocalize under focal adhesion forming conditions for cells containing overexpressed HER2 and beta-1-integrin [18]. The current hypothesis is that HER2 is expelled from focal adhesions for endogenous protein levels but the opposite happens for overexpression where the equilibrium between focal adhesions, HER2, and integrin levels is disrupted. However, experimental evidence is lacking for the hypothesized expelling which would manifest as anti-correlation, and moreover, integrin is not the most optimal marker for focal adhesions since the protein resides in the plasma membrane also in regions outside focal adhesions.

**Fig 1:**
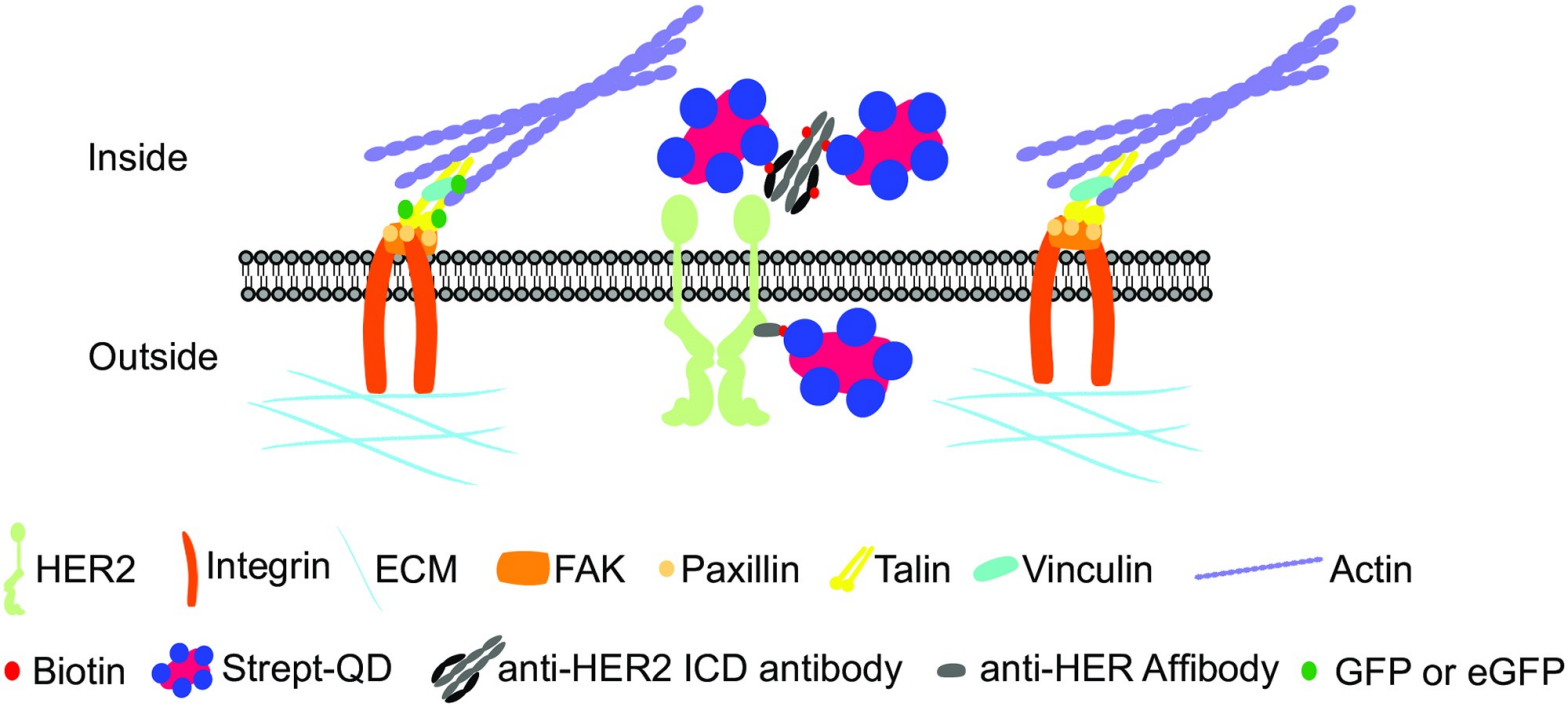
Labeling approaches for focal adhesions and HER2 used in this study. Focal adhesion complexes are formed once integrins (orange) bind to the extracellular matrix (cyan). Focal adhesion complex proteins in the cytoplasm (inside, upper part) connect the focal adhesion to the actin cytoskeleton (purple). Human epidermal growth factor receptor 2 (HER2) (pale green, indicated as a receptor dimer) is located in the membrane near focal adhesions and may interact with members thereof. For extracellular labeling of HER2 (outside, lower part), a biotin (small red) conjugated anti-HER2 affibody was used. For intracellular labeling of HER2 (inside, upper part), an anti-HER2 biotinylated antibody directed against the intracellular domain of HER2 was used. The primary biotinylated label was linked to a streptavidin (blue)-coated quantum dot nanoparticle (strept-QD, magenta). Note that for the affibody, one biotin binding site is available, whereas the antibody carries multiple biotins. Expression of green fluorescent protein (GFP) or enhanced green fluorescent protein (eGFP) (green) fused talin or vinculin was used to mark focal adhesion spots.

To study the possible spatial correlation or absence of correlation of focal adhesions and HER2 directly at the cell-substrate interface, the expression of talin- GFP or enhanced GFP (eGFP)-vinculin [21, 22] was used as a marker for functional focal adhesions along with extracellular or intracellular labeling of HER2 (Fig 1). Labeling of HER2 was achieved by either attaching a HER2 specific biotinylated affibody extracellularly [23] or a biotinylated antibody intracellularly. In both cases, streptavidin coated fluorescent QDs (strept-QDs) were added as the final step. QDs are non-organic labeling dyes that exhibit increased photo-stability compared to organic dyes, are small enough for tissue penetration, and allow for correlative light and electron microscopy (CLEM) [24]. Experiments were carried out using SKBR3 breast cancer cells overexpressing HER2 seeded on fibronectin and polyL-lysine coated glass substrates or silicon microchips for electron microscopy. Cells were analyzed by wide field fluorescence microscopy, total internal reflection fluorescent microscopy (TIRF), and scanning transmission electron microscopy (STEM) for an abundance of spatial colocalization of HER2 and focal adhesion members.

## Material and methods

### Materials and chemicals were purchased from the following companies

Biotin conjugated anti-HER2 Affibody (ZHER2:342)2 (from Affibody AB, Bromma, Sweden). Dulbecco’s phosphate-buffered saline (DPBS) (from Lonza, Cologne, Germany). Non-essential amino acids (NEEAs) 100x solution, strept-QD (Qdot^®^ 655 nm) streptavidin conjugate, Dulbecco’s Modified Eagle Medium (DMEM) GlutaMAX (high glucose and pyruvate), fetal bovine serum (FBS), BacMam talin-GFP, endogenous biotin blocking kit, anti-HER2 biotin monoclonal antibody (#MA5-13102) (all from Thermo Fisher Scientific GmbH, Dreieich, Germany). High pressure liquid chromatography (HPLC) grade double distilled H_2_O, acetone, ethanol, phosphate buffered saline (PBS) 1x solution, D-saccharose, sodium chloride, glycine, biotin free and molecular biology grade bovine serum albumin fraction V (BSA) and sodium cacodylate trihydrate (CB) (all from Carl Roth GmbH + Co. KG, Karlsruhe, Germany). Poly-L-lysine (hydrobromide (mol wt 70,000–150,000), sodium tetraborate, boric acid, TritonX-100, electron microscopy grade 25% glutaraldehyde solution, Xtreme Gene HP DNA transfection reagent (all from Sigma-Aldrich, Munich, Germany). Electron microscopy grade formaldehyde 16% solution (from Science Services GmbH, Munich, Germany). Normal goat serum (GS) (from Rockland Immunochemicals, Gilbertsville, PA, USA). 4-well, 35 mm compartments glass bottom dishes, and 96-well plates for tissue culture (from Greiner Bio-One GmbH, Frickenhausen, Germany). Silicon microchips with an electron transparent silicon nitride (SiN) window with dimensions of 175x400 μm^2^ (DENSsolutions, Delft, Netherlands). Multi-layer graphene poly(methyl methacrylate) was used (trivial transfer graphene, ACS Materials, Pasadena, CA USA).

### Mammalian cell culture

Human HER2-expressing breast cancer SKBR3 (HTB-30) cells (ATCC, Wesel, Germany) were kept at 37 °C, 5% CO_2_, and saturated water atmosphere in DMEM GlutaMAX (high glucose and pyruvate) supplemented with 10% FBS and 1% NEAAs. Cells were passaged twice a week by dissociation with CellStripper (Corning, Gernsheim, Germany), and kept at 60–80% confluency. Cells were not used beyond 20 passages. All cell cultures were tested for mycoplasma contamination every three months (Multiplexion, Heidelberg, Germany).

### Coating cell culture microscope dishes and microchips

Si Microchips (DENSsolutions, Netherlands) with electron transparent SiN windows (175 μm x 400 μm x 50 nm in size and thickness) were carefully removed from gel packs and rinsed in 100% HPLC-grade acetone for 2 min followed by a 2 min rinse in 100% HPLC-grade ethanol. Microchips were then washed for 5 min in H_2_O dipped to 100% HPLC-grade ethanol, and dried on a lint-free cleaning cloth. Labeling experiments of SKBR3 cells for light microscopy were carried out in 35 mm diameter, glass-bottom cell culture dishes with four-well compartments. Microchips or dishes were cleaned for 5 min with argon and oxygen in a plasma cleaner (Gatan, Munich, Germany), then incubated with 0.01% poly-L-lysine in water for 5 min. After two washes with double-distilled H_2_O, microchips or dishes were incubated with a solution of 15 μg/mL fibronectin-like protein in DPBS for 5 min, followed by two washing steps with 1x PBS. All steps were performed at room temperature. Microchips or dishes were covered with serum-free DMEM and kept at 37 °C with 5% CO_2_ until cells were seeded.

### Cell seeding on microchips or cell culture microscope dishes

For light microscopy, 80,000–100,000 cells per well were seeded in a pre-coated, 4-well compartment, glass-bottom cell culture dish. For STEM, 30,000 cells in a volume of 100 μL medium were added onto each pre-coated Si microchip in a 96-well plate compartment and incubated for 5 min at 37 °C. If necessary, more cells were added until approximately 30–60 cells adhered to the SiN window. Cells were grown for 24 hours in 10% FBS DMEM, after which the cells were transduced with BacMam talin-GFP.

### Transduction and transfection of focal adhesion markers

Transduction of BacMam talin-GFP (ThermoFisher #C10611) was carried out according the manufacturer’s instructions. In brief, for transduction of 4-well compartment dishes, 10 μL of BacMam talin-GFP was used in a total volume of 300 μL. For cells grown on microchips, transduction was carried out in a 48-well plate using 10 μL BacMam Talin-GFP in a total volume of 300 μL. Transduction took place overnight (16 hours). The cells were then starved for 4 hours in serum-free DMEM (SF-DMEM) to enhance the amount of HER2 in the plasma membrane. For vinculin transfection was carried out using a pcDNA3 vector encoding for the eGFP-vinculin fusion protein (kindly provided by Johan de Rooij, University Medical Center Utrecht, Netherlands) [22]. For transfection, 1x10^5^ SKBR3 cells were seeded per 6-well plate compartment and grown overnight. The growth medium was changed to serum-free medium the next day. For each well, a total of 2 μg of pcDNA3 eGFP-vinculin plasmid DNA was diluted in 500 μL serum-free medium, and mixed with 6 μL of transfection agent (Xtreme Gene HP DNA transfection reagent (Sigma-Aldrich #06366244001)). The mixture was incubated for 15 min at room temperature, then added to cells dropwise. Medium was exchanged after 3–6 hours to 10% FBS medium, in which the cells were incubated for 24 hours.

### Extracellular labeling of HER2 on dishes

For HER2 labeling, cells were transferred to pre-coated 4-well compartment, glass bottom microscopy dishes after transfection (for eGFP-vinculin), and allowed to attach for 24 hours. The cells were then starved for 16 hours in serum-free medium. Talin-GFP expressing cells were starved for 4 hours after overnight transduction as described above. After starvation, extracellular labeling of HER2 was carried out similar as described elsewhere [23]. Reagents were used in the following concentrations: Biotinylated anti-HER2 affibody, 200 nM diluted in blocking solution (serum-free DMEM (SF-DMEM) containing 1% BSA and 1% goat serum); strept-QD), 1 μM stock solution was centrifuged for 4 min at 8,000 g, and a final concentration of 5 nM was achieved by pre-diluting stock 1:3 in 40 mM borate buffer pH 8.3 to avoid strept-QD clumping, and further diluted in PBS with 1% BSA to the final concentration. All solutions were warmed up to 37 °C for steps before fixation or room temperature for all steps during and after fixation. Cells were washed with SF-DMEM once followed by a 5-minute blocking step at 37 °C. Thereafter, cells were incubated with the biotinylated anti-HER2 affibody for 10 min at 37 °C, washed with SF-DMEM twice, and once with CB (0.1 M cacodylate buffer, 0.1 M sucrose pH 7.4) before being fixed in 3% formaldehyde (electron microscopy grade) diluted in CB for 10 min at room temperature. After additional washing steps (1x CB and 3x PBS), cells were quenched with 0.1 M glycine-PBS for 2 min, rinsed with PBS once, and incubated with 5 nM stept-QD for 12 min at room temperature. After three washing steps with PBS/ 1% BSA, the cells were covered with PBS/ 1% BSA for subsequent fluorescence microscopy analysis.

### Intracellular labeling of HER2 on dishes and microchips

For intracellular labeling of HER2, SKBR3 cells were washed with PBS, fixed in 3% formaldehyde diluted in CB for 10 min at room temperature. After three washing steps with PBS, cells were permeabilized and blocked with PBS containing 0.3% surfactant (TritonX-100) and 5% goat serum for 1 hour at room temperature. Endogenous biotin was blocked (2x 15 min) using an endogenous biotin blocking kit (ThermoFisher #E21390) before HER2 was labeled with anti-HER2 biotin antibody (ThermoFisher #MA5-13102) 1:200 in PBS containing 1% BSA and 0.3% surfactant for 1 hour at room temperature. Cells were washed with PBS/ 1% BSA three times, incubated with 20 nM strept-QD (prepared as above) for 12 min at room temperature, and washed with PBS/ 1% BSA three times. Cells were covered with PBS/ 1% BSA for fluorescence microscopy. For imaging Si microchips, these were placed upside-down in 35 mm glass-bottom dishes in 1mL PBS/ 1% BSA. After imaging cells grown on microchips were further post-fixed with 2% glutaraldehyde (GA) (EM-grade). First, the microchips were washed with CB once, fixed with 2% GA for 10 min at room temperature, washed with CB once, and with PBS thrice. Thereafter, microchips were stored in PBS/ 1% BSA/ 0.02% NaN_3_ until electron microscopic analysis.

### Direct interference contrast and fluorescence microscopy

Labeled cells were imaged with an inverse microscope (DMi6000B, Leica) using a 63x (HC PL APO 63x/1.40 oil) objective. The following channels were acquired: direct interference contrast (DIC), fluorescence optimized for GFP (filter L5 ex. 460–500 nm/ em. 512–542 nm), and fluorescence optimized for strept-QD (filter TX2 ex. 540–580 nm/ em. 607–683 nm). Multiple images with a size of 1,392x1,040 pixels each and focal series were automatically recorded (Leica DFC365 FX camera and LAS FX software version 3.4.1.17822).

Alternatively, images were taken with the ZEISS Observer Z1 with a 40x (Plan-Apochromat 40x/1.4 Oil) objective using multi-channel acquisition for phase (DIC), GFP (filter 38 GFP ex. 450–490 nm/em.500-550 nm) and strept-QD (filter 62 HE BFP/GFP/HcRed ex. 567–602 nm/em.615-4095 nm). Images were acquired with the ZEISS software, ZenBlue, with a connected AxioCam 506 with 2,752x2,208-pixel resolution.

### TIRF microscopy

TIRF microscopy was performed using a 100x/ 1.49 oil Apochromat TIRF objective with a connected TI-TIRF-E motorized illuminator (Nikon, Eclipse TI-E microscope, Andor DU-888 X-11105 EMCCD camera, NIS Elements software version 4.60.00 with the Ti-LAPP module). Images were acquired with a resolution of 1,024x1,024 pixels and 16-bit bit-depth. The 488 nm laser was used for GFP images, and the 638 nm laser was used for strept-QD, both with the quad-band TIRF filter set (TRF89901—Chroma).

### Quantitative fluorescence measurements

Fluorescence images for GFP and strept-QD were analyzed with Fiji ImageJ (Ver.1.51) (NIH) using the provided measure tool. The region of interest (ROI) located at talin spots or HER2-enriched regions, and also background spots outside cells were defined manually in 16-bit grayscale images. The area, the mean gray value, and the integrated fluorescence intensity (indicated as integrated density in ImageJ) in a ROI were analyzed for each defined spot in the images. To determine the corrected integrated fluorescence intensity (CFI) of a ROI, the following formula was applied:

CFI=IntegratedintensityinROI-(areaofROI*meanbackgroundfluorescenceintensity)

whereby the mean background fluorescence intensity was obtained from the mean gray value in a ROI outside the cell. CFI values were compared and statistically analyzed.

### Graphene enclosure of microchips for electron microscopy

Multilayer graphene sheets were transferred to NaCl crystals as described previously [25]. To obtain graphene floating on water, a piece of NaCl crystal carrying enough graphene to cover the SiN window of the microchip was dipped into H_2_0 at a 45° angle to detach the graphene layer from the salt crystal. The free-floating graphene was then captured with a metal loop so that the graphene was floating in a water droplet stuck in the metal loop. The microchip to be covered was washed in H_2_0 thrice to remove PBS/ 1% BSA/ 0.02% NaN_3_, and was then attached to the lower surface of the metal loop with the graphene floating in the droplet of water above. Excess water was removed by filter paper under a binocular, leaving the cells hydrated under the multilayer graphene.

### Scanning transmission electron microscopy (STEM)

Microchips with graphene coated cells were mounted on a standard holder (JEOL), and imaged with a transmission electron microscope (JEM-ARM 200F, JEOL) operated in STEM mode. The microscope was equipped with a cold field emission gun, and a STEM probe corrector (CEOS GmbH), and was operated at 200 keV. Images were acquired with a condenser lens aperture of 20 μm, a probe current of 175 pA, a pixel dwell time of 6 μs. The electron beam was set to a beam convergence angle of 13.2 mrad and the STEM detector opening angle β_in_−β_out_ = 68–280 mrad. The image size was 2,048x2,048 pixels. Overview images were acquired in low magnification mode of 800x (pixel size of 0.13 μm), while magnified images were acquired at 20,000x and 60,000x magnification (pixel sizes of 5 nm or 1.7 nm, respectively) with the Gatan software Digital Micrograph (Ver. 3.30.2016.0). The maximal electron dose applied at 60,000x magnification amounted to 23 e^-^Å^-2^.

### Particle analysis

To measure the number of labeled HER2 proteins in an image, the strept-QDss in a STEM image were automatically detected using a plugin of local design [23] for Fiji ImageJ (Ver1.52) (NIH). The plugin functioned as follows, STEM images were first subjected to a Gaussian filter with a radius of 1.5 pixels for noise filtering. Then a Fourier filter was applied to negate variations in the image background, followed by binarization of the image with an automatic threshold with maximal entropy setting. For the detection of strept-QDs, the “analyze particle” tool was applied with settings of a particle size of 11 nm ± 2 nm, a total number of 100 bins, and a bin width of 3 nm. Strept-QD densities for each image were calculated by dividing the number of strept-QDs per image by the analyzed image area, and results were analyzed statistically.

### Statistical analysis

CFI values of fluorescent images, and STEM image derived strept-QD densities were analyzed using GraphpadPrism7.0. Data sets were compared using an unpaired two-tailed t-test. A p-value less than 0.05 was considered statistically significant. Box plot graphs show min to max representations with indicated median value and standard deviation, and each dot representing one CFI value or strept-QD density measurement.

## Results and discussion

### Co-labeling of focal adhesions and HER2 on SKBR3 cells

**Fig 2:**
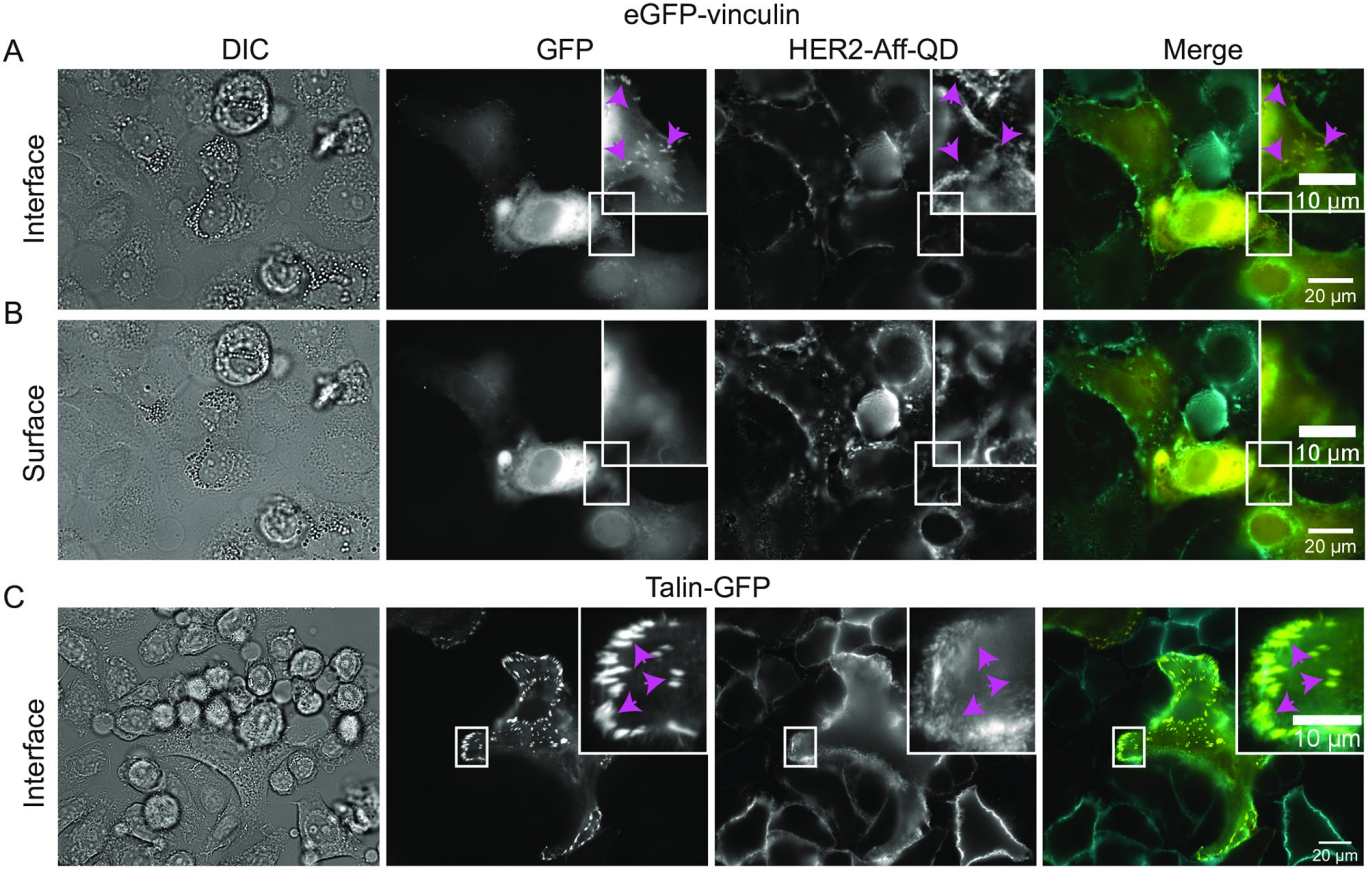
Co-labeling of focal adhesion markers vinculin or talin with HER2. Direct interference contrast (DIC) and wide field fluorescence images of SKBR3 cells seeded on glass-bottom dishes. The cells were either transfected with eGFP-vinculin (A and B) or transduced with talin- GFP (C) to label focal adhesion spots (GFP signal, magenta arrow heads). HER2 was labeled by biotinylated anti-HER2 affibody coupled to strept-QD (HER2-Aff-QD). (A) The focus was adjusted to the basal side of the cells at the interface with the substrate. Images were acquired using a 63x objective. (B) Similar images as in (A), but with the focus adjusted to apical side of the cell. (C) Images of SKBR3 cells with labeled HER2 and transfected with talin-GFP. Images were acquired using a 40x objective. Colors in merged images: yellow for GFP and cyan for HER2-Aff-QD. Scale bars: 20 μm and 10 μm for the insets.

To analyze HER2 expression within the proximity of focal adhesions, a labeling protocol was developed for SKBR3 breast cancer cells. Focal adhesions were tagged by transfection of eGFP-vinculin (Fig 2A and 2B) that allows labeling of integral proteins of the adhesion complex [22]. Focal adhesions were visible as bright spots at the bottom side of the cell at the interface with the glass substrate (Fig 2A). Most were located at the outer edges of the cells as bright spots (Fig 2A, see also S1A and S1B Fig for cells grown at lower density), but some were also visible within the contour underneath the cell (Fig 2A inset). Towards the apical surface of the cells, the GFP fluorescence signal disappeared (Fig 2B), indicating that the focal adhesion complexes were arranged at the cell-surface interface as expected [26] (Fig 2A and S1 Movie).

Co-labeling of HER2 was applied to determine if HER2 would enrich at focal adhesion spots. HER2 was extracellularly labeled with a biotinylated-anti-HER2-affibody linked to a strept-QD as described elsewhere [23]. In short, HER2 was first linked to the anti-HER2 biotinylated affibody. Cells were then fixed before strept-QDs were coupled to the biotinylated affibodies. This two-step labeling procedure was applied to prevent labeling-induced receptor clustering that may occur when the affibody is linked to the strept-QD first because the strept-QD has multiple streptavidins on its surfaces. QD fluorescence was present at the cell edges (Fig 2A and S1A and S1B Fig) as well as at higher-up regions throughout the cell surface with local enrichments in clusters on membrane ruffles (Fig 2B areas of brighter fluorescence intensity). The QD fluorescence intensity was low at the cell-surface interface below the cell (see also S2 Movie) indicating that most attached HER2 labels were located above that plane. To analyze the expression of HER2 in the spatial proximity of focal adhesions, the fluorescence patterns were analyzed at the edges of flattened cells, regions where both signals were derived from similar focal planes (insets Fig 2A and S1B Fig). The spatial patterns of higher and lower intensity did not overlap between QD and GFP fluorescence. Although HER2 was expressed in the areas containing focal adhesions at the cell edges, the density of HER2 was not enriched at the focal adhesion spots (Fig 2A and 2C magenta arrowheads and insets S1B Fig). Thus, there does not seem to be a colocalization. Colocalization neither was found in any other analyzed cell (Table 1). To rule out the possibility that the studied colocalization depended on the type of protein in the focal adhesion, the colocalization of HER2 was tested with talin, another protein of focal adhesions [21] (Fig 2C and S1C and S1D Fig). With the talin-GFP system, a similar distribution of focal adhesion spots was observed. Most focal adhesion spots were located at the cell periphery of the cell-substrate interface (Fig 2C and S1C and S1D Fig) and colocalization of talin with HER2 was not observed (insets Fig 2C and 2D and S1D Fig, Table 1). Instead, an anti-correlated expression of high talin and low HER2 expression was observed at cell periphery (S1C and S1D Fig and S3 Movie).

**Table 1:** Overview of analyzed data. Information is provided about the labeling method, the microscopy method, the total number of analyzed images, the number of analyzed cells, and the amount of focal adhesion marker expressing cells. Figs 2–4 show representative images for each experiment (see also related S1–S3 Figs).

Figure, Focal Adhesion Marker	HER2 labeling method, microscopy method, magnification	Analyzed Images	Analyzed cells	Focal adhesion marker positive cells	Comments
Fig 2A and 2B S1A and S1B Fig	Extracellular HER2 labeling.	4	31	9	Low transfection efficacy for eGFP-vinculin.
	Wide field fluorescence microscopy				Different focal areas for thick areas of cells impede colocalization analysis.
Vinculin	630x				Only for flattened areas at the cell edge colocalization could be analyzed. No overlapping expression was observed for vinculin and HER2.
Fig 2C	Extracellular HER2 labeling.	7	156	59	Colocalization analysis was impaired by different focal planes. No overlapping expression was observed for talin and HER2.
S1C and S1D Fig	Wide field fluorescence microscopy				
Talin	400x				
Fig 3A and 3B	Intracellular HER2.	10	64	34	TIRF analysis of intracellular labeled of HER2 confirmed HER2 expression at the cell-substrate interface. Talin was mainly expressed in HER2-low regions.
	TIRF				
S2 and S3 Figs	1000x				
Talin					
Fig 3C	Intracellular HER2.	9	46	32	Control experiment omitting the HER2 antibody for intracellular labeling.
	TIRF				
Talin	1000x				Almost no unspecific binding of strept-QDs was observed.
Fig 3D and 3E	Quantification of CFIs	5	16	16	HER2 expression intensities were decreased at talin positive spots.
Fig 4	Intracellular HER2 labeling	9	34	19	Wide field fluorescence microscopy revealed HER2 expression at the cell substrate interface. HER2 expression was heterogeneous with HER2-low and HER2-enriched regions. Talin expression was mainly observed in HER2-low regions.
	Wide field fluorescence				
Talin	microscopy				
	630x				
Fig 4	Intracellular HER2 labeling	28	10	2	STEM revealed that HER2-QDs were mostly found outside talin-GFP positive regions.
Talin	STEM				
	20,000x or 60,000x				

### Studying colocalization of talin and HER2 via intracellular HER2 labeling

The lack of colocalization may have resulted from the labeling procedure rather than from a biological factor, since labeling of HER2 at the cell-surface interface may have been hindered by steric factors. Therefore, an alternative labeling approach for HER2 receptors was developed in which the HER2 receptors were targeted with QDs via the intracellular domain (Fig 1). Talin-GFP was used for this experiment, since it exhibits a higher labeling efficacy than vinculin in terms of transduced cells and fluorescence intensity at focal adhesions (see Fig 2A and 2C GFP channel). Focal adhesion spots were labeled first by transduction of talin-GFP, after which the cells were fixed and the cell membrane was permeabilized. The endogenous biotin was masked before HER2 was labeled with a biotinylated antibody directed against the intracellular C-terminal part of HER2. The strept-QD component was attached at last. The obtained label was thus biotinylated anti-HER2 antibody coupled to strept-QD (HER2-QD). TIRF microscopy was used to selectively visualize fluorescent labels in the evanescent field (30–100 nm) directly above the glass substrate. TIRF thus allowed imaging of focal adhesions and QDs bound to HER2 expressed at the cell-substrate interface without interfering fluorescence signal derived from non-focal planes.

**Fig 3:**
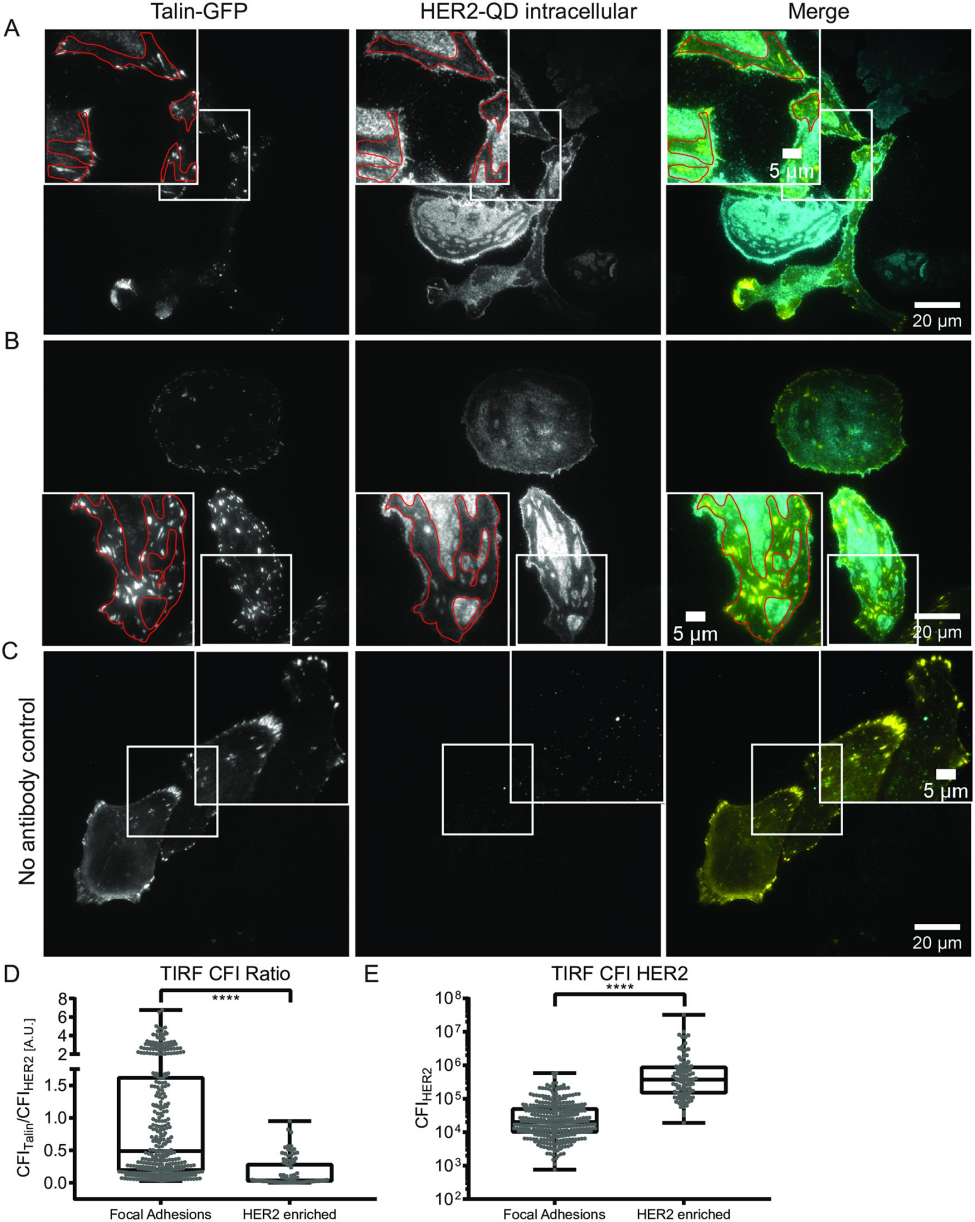
Analysis of intracellular HER2 labeling and co-labeling with talin confirms HER2 expression at the cell substrate interface. (A and B) Total internal reflection fluorescence (TIRF) microscopy of SKBR3 cells transduced with talin-GFP on glass-bottom dishes analyzed with a 100x oil TIRF optimized objective. The intracellular domain of HER2 was labeled with strept-QD via biotinylated anti-HER2 antibody coupled to strept-QD (HER2-QD). Outlined regions indicate HER2-low expression areas. (C) Control experiment using the same protocol as A and B, but without anti-HER2 antibody. (D) Box plot graph displaying the ratios of talin-GFP to HER2-QD corrected integrated fluorescence intensities (CFI) measured at focal adhesions (left) and HER2-enriched regions (right). (E) Box plot graph displaying the CFI for HER2-QD measured at focal adhesions or at HER2-enriched regions. (D, E) **** for p < 0.001, unpaired, two-tailed t-test, n = 339 for talin spots and n = 95 for HER2-enriched regions, obtained from 16 different cells expressing variable amounts of talin. Box plot graphs show min to max representations with indicated median value with each point representing one measurement. Colors in merged images: yellow for GFP and cyan for strept-QD. Scale bars: 20 μm and 5 μm for insets.

Fig 3 shows exemplary images of intracellularly labeled HER2 in talin-GFP transduced SKBR3 cells (see S2 and S3 Figs for full images including DIC). Note that the focus was set to the cell surface interface displaying the talin-GPF positive focal adhesion spots (Fig 3A–3C left). Here, at the cell substrate interface, the QD fluorescence showed a different pattern compared to the extracellularly labeled HER2 (Fig 2) with clearly defined HER2 expression patterns (enriched and low regions) (Fig 3A and 3B middle). A control experiment omitting the HER2 antibody reported almost no unspecific label for the strept-QDs (Fig 3C). Interestingly, talin-GFP expression was mostly observed in the HER2-low regions (Fig 3A and 3B, right and red outlined areas), indicating an anti-correlative expression. CFI measurements for talin spots and HER2-enriched regions confirmed this finding. The CFI ratio of talin-GFP toHER2-QD was higher at focal adhesion compared to HER2-enriched regions (Fig 3D). In contrast, the HER2-QD CFI was significantly higher for HER2-enriched regions compared to talin-GFP spots (Fig 3E). This observation did not depend on amount of the talin-overexpression. S3 Fig shows an exemplary quantification of the CFI ratios of the two different cells presented in Fig 3B, and indeed this revealed similar results for the talin high expressing cell (lower cell in Fig 3B) and the talin low expressing cell (upper cell in Fig 3B). These results show that HER2 is heterogeneously localized at the cell-substrate interface, and that regions of high HER2 localization do not overlap with focal adhesion localization.

### Correlative light- and electron microscopy confirmed an anti-correlative expression of HER2 and talin

**Fig 4:**
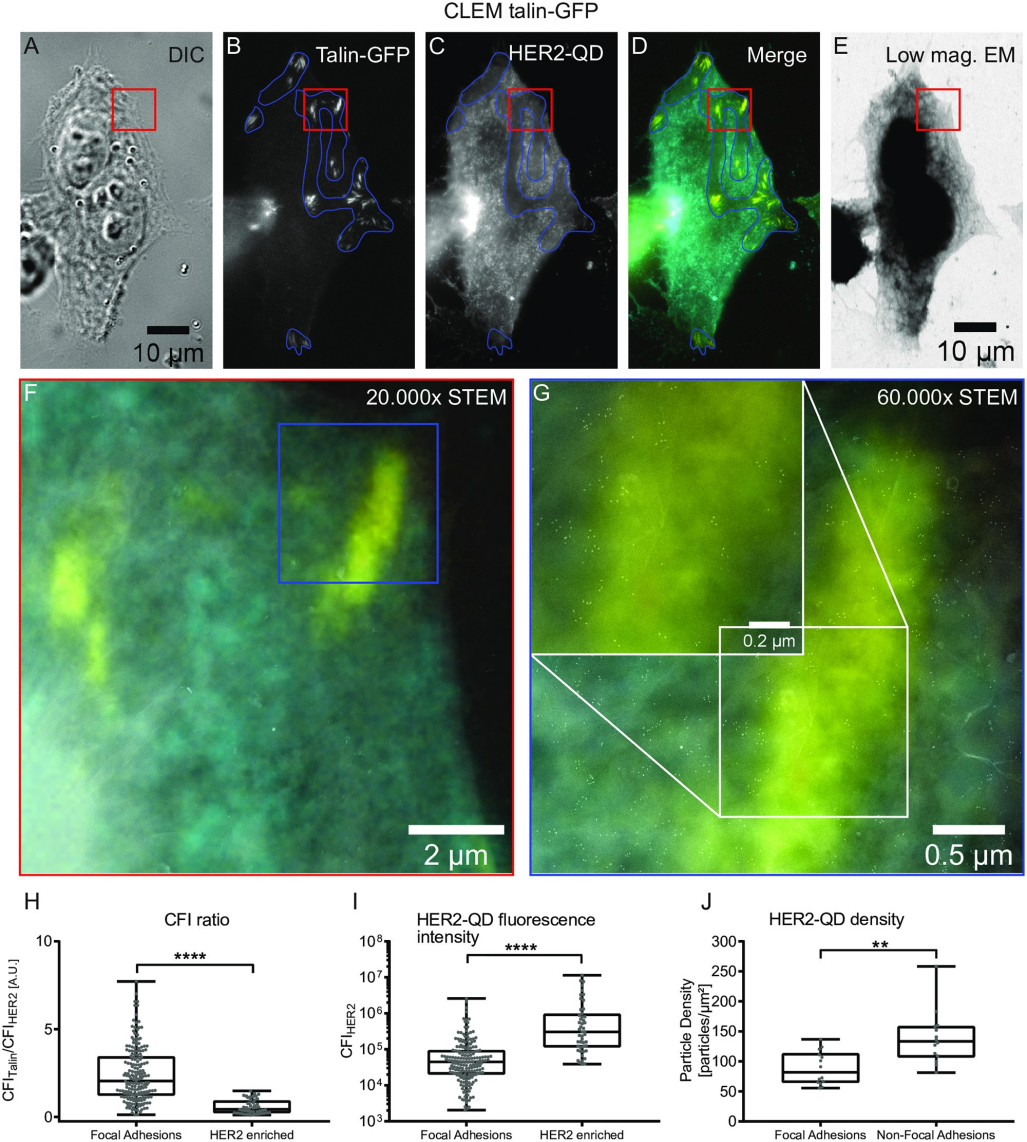
Correlative light- and electron microscopy (CLEM) of intracellularly HER2-QD, and talin-GFP at focal adhesions. (A-E) Example image of a SKBR3 cell grown on a silicon nitride window of a silicon microchip suitable for correlative light- and electron microscopy. Shown are DIC (A), fluorescence images for talin-GFP (B), HER2-QD (C), the fluorescent channel overlay image (D), and an 800x low magnification (low mag.) bright field scanning transmission electron microscopy (STEM) image (E) of the same cell. HER2 low regions are outlined in blue. The rectangle outlined in red indicates the magnified region shown in (F) acquired with dark field STEM and overlaid with the corresponding magnified merge fluorescent image. (G) displays a zoom of the highlighted region in blue in (F). A further magnified region is shown in the inlet (white rectangle). Note that the talin-positive region shows a lower abundance of HER2-QDs (yellow stripe). (H) Box plot graph displaying the ratios of talin-GFP to HER-QD CFIs measured at focal adhesions (left) and HER2-enriched regions (right). (I) Box plot graph displaying the CFI for HER2-QD measured at focal adhesions or at HER2-enriched regions. **** for p < 0.001, unpaired, two-tailed t-test, n = 192 for talin spots and n = 48 for HER2-enriched regions. (J) Box plot graph displaying the HER2-QD density measured from the STEM image for focal adhesion versus non-focal adhesion regions. ** for p < 0.01 unpaired, two-tailed t-test, n = 14 for both talin spots and non-talin spots. Graphs display min to max representations with indicated median value. Each point represents one CFI measurement (H and I) or the particle density of one analyzed image (J). Colors in merged images: yellow for GFP and cyan for HER2-QD. Scale bars: 10 μm in (A), 2 μm (F), 0.5 μm and 0.2μm for (G).

To confirm our finding that HER2 and talin expression are anti-correlated, the expression of the HER2 receptor with the intracellular HER2-QD label was analyzed at focal adhesions with STEM at the nanometer scale. To this end, SKBR3 cells were seeded on poly-L-lysine and fibronectin coated silicon microchips with an electron beam transparent silicon nitride window. After transduction of talin-GFP and intracellular labeling of HER2, cells were analyzed by wide field fluorescence light microscopy (Fig 4A–4D). As seen before (Fig 3), HER2 expression was divided in HER2-enriched and HER2-low regions (Fig 4C), and talin-GFP was predominately observed in those HER2-low regions (Fig 4D and blue outlined regions). CFI ratios of talin-GFP to HER2-QD, and the CFI for HER2-QD at the talin spot or HER2-enriched region again confirmed lower HER2 expression at talin-positive focal adhesion spots (Fig 4H and 4I). For subsequent STEM analysis, cells were covered with 3–5 multi-layered graphene to protect them from vacuum induced damaging and to keep the cells in a hydrate state. Fig 4E shows the region corresponding to the light microscopy images acquired in bright field low magnification STEM mode. Fig 4F displays the dark field acquired high magnification STEM image of the red outlined region in Fig 4A–4E. Individual strept-QDs became visible after a further magnification (Fig 4G bright spots and magnified regions within Fig 4G). The corresponding magnified fluorescent merged image (Fig 4D) was used to overlay both dark field acquired STEM images (Fig 4F and 4G) to track focal adhesion spots (yellow). Most QDs were present outside these regions. In contrast to extracellular labeling for which one HER2 receptor was bound by one biotin-HER2 affibody coupled to one strept-QD (1:1:1 ratio) (Fig 1 and [23]), the intracellular labeling with intracellular domain (ICD) anti-HER2 biotin antibody provided up to six binding sites for strept-QD (1:1:1–6 ratio) (Fig 1). Therefore, the observed clustering of QDs in Fig 4G does not necessarily report HER2 protein clusters. Nonetheless, HER2 distribution analysis at focal adhesion areas, identified by the overlaid fluorescent images, was possible and revealed that less QDs were present at talin spots compared to areas outside talin spots (Fig 4G and quantification Fig 4J).

This data showing anti-correlation between HER2 and focal adhesions confirms the hypothetical model described by Toscani et al. in which HER2 is expelled from focal adhesions in the plasma membranes of adherent cells at endogenous protein levels [18]. Activated focal adhesions might thus form micro-domains in the membrane from which HER2 receptors are excluded. In contrast to the cell surface expression of HER2 that is homogenous, albeit with variations in the expression level for different cells and with enrichments in membrane ruffles (Fig 2, extracellular label), the HER2 expression at the cell substrate interface thus clearly divides in HER2-low and -enriched regions.

## Conclusion

An intracellular labeling protocol was developed for CLEM that allows for protein analysis within intact cells demonstrated for HER2 expression analysis at focal adhesion spots. This approach can also be used for studying other membrane proteins that are not accessible by extracellular labeling. The above experimental findings show that HER2 and focal adhesion proteins, talin or vinculin, did not colocalize on breast cancer cells on fibronectin and poly-L-lysine coated substrates. The expression of HER2 at the cell-substrate interface was heterogeneous and divided into HER2-low, and HER2-enriched regions. Focal adhesions were found preferentially in areas of HER2-low expression areas and *vice versa*, showing an anti-correlative expression of focal adhesions and HER2 on adherent SKBR3 breast cancer cells. A functional mechanism may, therefore, exist that involves a lower amount of HER2 at a focal adhesion to support the adherence of a migrated cancer cell to an extracellular matrix in the process of metastasis formation.

## Supporting information

S1 Fig Co-labeling of focal adhesion markers vinculin or talin with HER2 (related to Fig 2). Direct interference contrast (DIC) and wide field fluorescence images of SKBR3 cells seeded on glass-bottom dishes. The cells were either transfected with enhanced green fluorescent protein (eGFP)-vinculin (A and B) or transduced with talin-green fluorescent protein (GFP) (C and D) to label focal adhesion spots (GFP signal, white boxes in B and D). Human epidermal growth factor receptor 2 (HER2) was labeled extracellularly by biotinylated anti-HER2 affibody coupled to streptavidin-quantum dot (strept-QD, HER2-Aff-QD). (A) Parts of a flattened, spread cell is shown. White rectangle indicates the magnified region in (B) highlighting focal adhesion spots (white squares). Image was acquired using a 63x objective. (C) Images of SKBR3 cells with labeled HER2 and transfected with talin-GFP. White rectangle in (C) indicates magnified region shown in (D) highlighting focal adhesion spots (white squares). Image was acquired using a 40x objective. Colors in merged images: yellow for GFP and cyan for HER2-Aff-QD. Scale bars: 20 μm and 5 μm for the insets. See also S3 Movie.
(PDF)

S2 Fig Total internal reflection fluorescence (TIRF) image of talin-GFP expressing cells with intracellularly labeled HER2 (related to Fig 3A). TIRF microscopy of SKBR3 cells transduced with talin-GFP on glass-bottom dishes analyzed with a 100x oil TIRF optimized objective. The intracellular domain of HER2 was labeled with a biotinylated ant-HER2 antibody coupled to strept-QD (HER2-QD). The same image as in Fig 3A is shown. The outline region indicates the magnified region shown Fig 3A. Shown are DIC, talin-GFP, HER2-QD fluorescence images and a merge image. Colors in merged image: yellow for GFP and cyan for HER2-QD. Scale bar: 20 μm.
(PDF)

S3 Fig Corrected fluorescence intensity (CFI) analysis of TIRF images (related to Fig 3B). (A) DIC, talin-GFP, HER2-QD fluorescence images and merge image of SKBR3 cells acquired with TIRF (same image as in Fig 3B). Manually marked talin spots for fluorescence intensity analysis (B) are highlighted in all images (yellow). (B) Comparison of CFI ratios of talin to HER2 for talin high expressing cell (lower cell in S3A Fig) and low expressing cell (upper cell in Fig 3A). Similar ratios are seen for talin high (left) and talin low (right) expression. Each point represents one CFI ratio. n = 67 for the talin high expressing cell, n = 46 for the talin low expressing cell. Note that this analysis is part of the overall analysis shown in Fig 3D. Colors in merged image: yellow for GFP and cyan for HER2-QD. Scale bar: 20 μm.
(PDF)

S1 Movie Fluorescence microscopy focal series channel corresponding to GFP vinculin. A focal series (Z-Stack) of 17 images was acquired from the apical surface to the cell surface interface with a 63x oil objective and a step size of 0.407 μm. This dataset was used for Fig 2A and 2B in the main text.
(AVI)

S2 Movie Fluorescence microscopy focal series channel corresponding to HER2-Aff-QD. A focal series (Z-Stack) of 17 images was acquired from the apical surface to the cell surface interface with a 63x oil objective and a step size of 0.407 μm. This dataset was used for Fig 2A and 2B in the main text.
(AVI)

S3 Movie Alternating fluorescent images of HER2-Aff-QD (grayscale) and HER2-Aff-QD with talin-GFP (merged). Talin-GFP expression (yellow) is mainly observed at the cell periphery where HER2 expression (cyan and grayscale, alternating)) is reduced. Image was acquired using a 40x objective and cropped. The same image is shown in S1C and S1D Fig. Colors in merged images: yellow for GFP and cyan for HER2-Aff-QD. Scale bar: 5 μm. This movie is related to Fig 2 and S1 Fig. The same two images are alternated for comparison of both fluorescence signals. Note the reduced expression of HER2 at talin positive spots.
(AVI)

## Abbreviations

BSA: bovine serum albumin
CB: sodium cacodylate trihydrate buffer
CFI: corrected integrated fluorescence intensity
DIC: direct interference contrast
DMEM: Dulbecco’s Modified Eagle Medium
DPBS: Dulbecco’s PBS
ECM: extracellular matrix
eGFP: enhanced GFP
EGFR: epidermal growth factor receptor
EMT: epithelial to mesenchymal transition
FAK: focal adhesion kinase
FBS: fetal bovine serum
FLP: fibronectin like protein
FN: fibronectin
GA: glutaraldehyde
GFP: green fluorescent protein
GS: goat serum
HER2: human epidermal growth factor receptor 2
HER2-Aff-QD: biotinylated anti-HER2 affibody coupled to strept-QD
HER2-QD: biotinylated anti-HER2 antibody coupled to strept-QD
HPLC: high pressure liquid chromatography
ICD: intracellular domain
NEAAs: non-essential amino acids
PBS: phosphate buffered saline
PI3K: Phosphoinositide 3-kinase
PLL: poly-L-lysine
QD: quantum dot
ROI: region of interest
SF-DMEM: serum-free DMEM
STEM: scanning transmission electron microscopy
strept-QD: streptavidin quantum dots
TIRF: total internal reflection fluorescent microscopy
