# Supplementary figures and images for: Anti-correlation of HER2 and focal adhesion complexes in the plasma membrane
Source: PLoS One. 2020 Jun 8;15(6):e0234430. doi: 10.1371/journal.pone.0234430 (PMC7279600; doi:10.1371/journal.pone.0234430)

# eGFP-vinculin

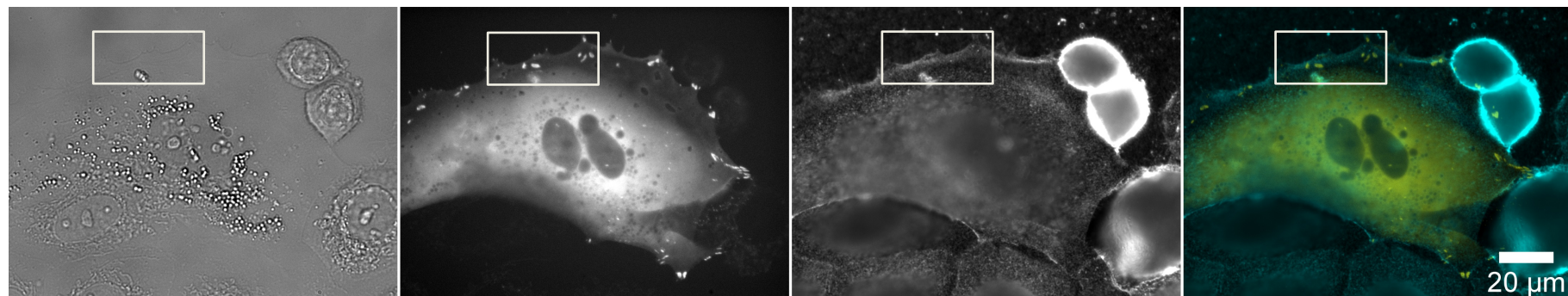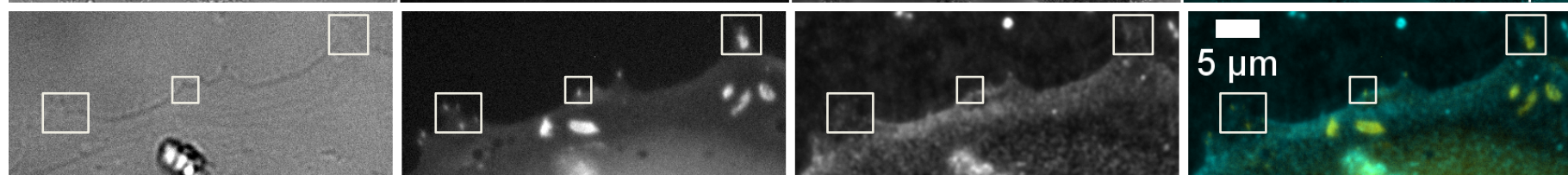

# Talin-GFP

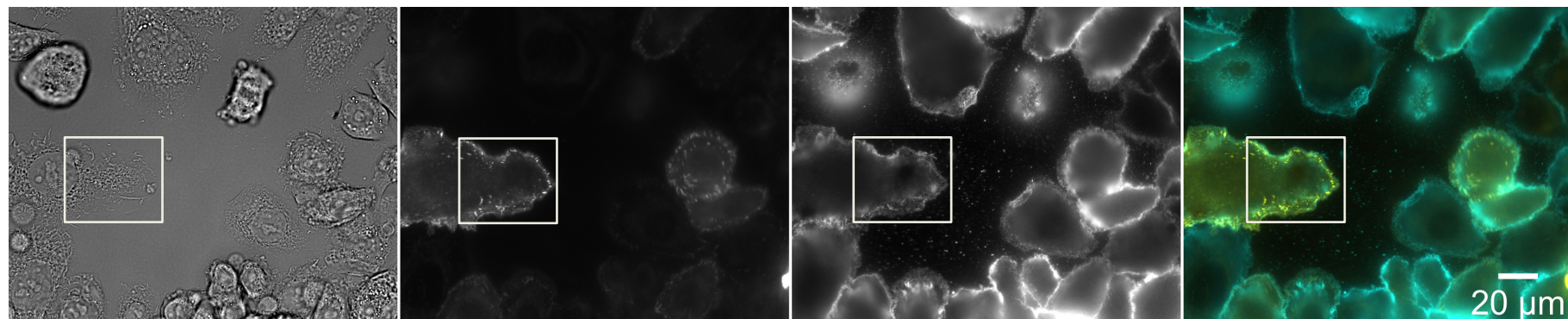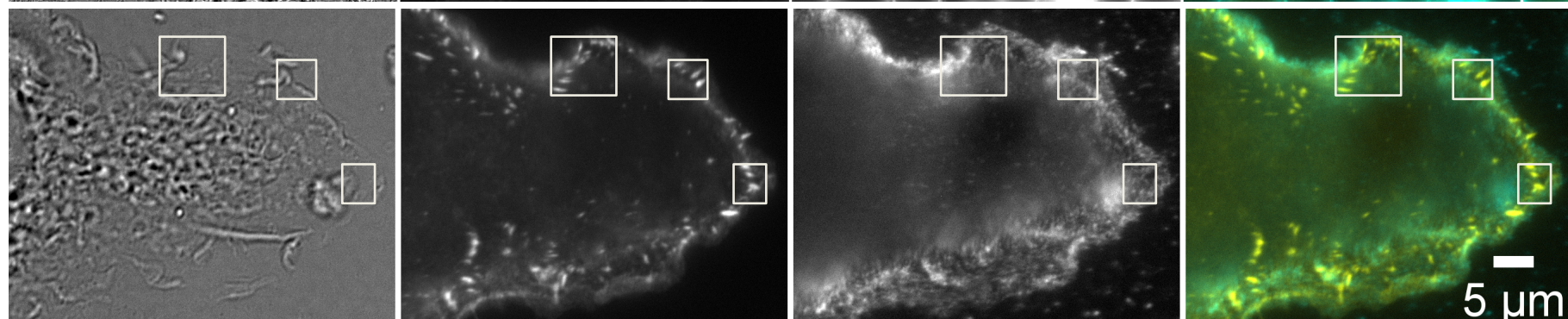

DIC

GFP

HER2-Aff-QD

Merge

Supplement: S1 Fig — Direct interference contrast (DIC) and wide field fluorescence images of SKBR3 cells seeded on glass-bottom dishes. The cells were either transfected with enhanced green fluorescent protein (eGFP)-vinculin (A and B) or transduced with talin-green fluorescent protein (GFP) (C and D) to label focal adhesion spots (GFP signal, white boxes in B and D). Human epidermal growth factor receptor 2 (HER2) was labeled extracellularly by biotinylated anti-HER2 affibody coupled to streptavidin-quantum dot (strept-QD, HER2-Aff-QD). (A) Parts of a flattened, spread cell is shown. White rectangle indicates the magnified region in (B) highlighting focal adhesion spots (white squares). Image was acquired using a 63x objective. (C) Images of SKBR3 cells with labeled HER2 and transfected with talin-GFP. White rectangle in (C) indicates magnified region shown in (D) highlighting focal adhesion spots (white squares). Image was acquired using a 40x objective. Colors in merged images: yellow for GFP and cyan for HER2-Aff-QD. Scale bars: 20 μm and 5 μm for the insets. See also S3 Movie. (PDF) [file pone.0234430.s001.pdf]

DIC

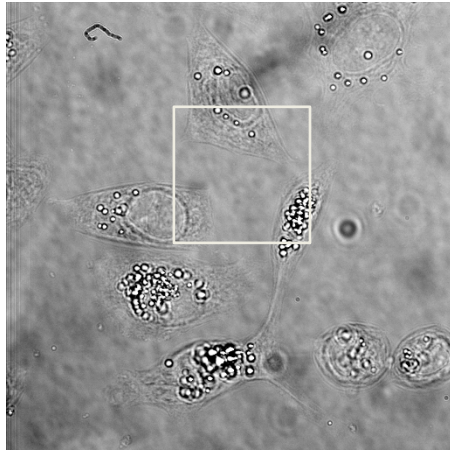

Talin-GFP

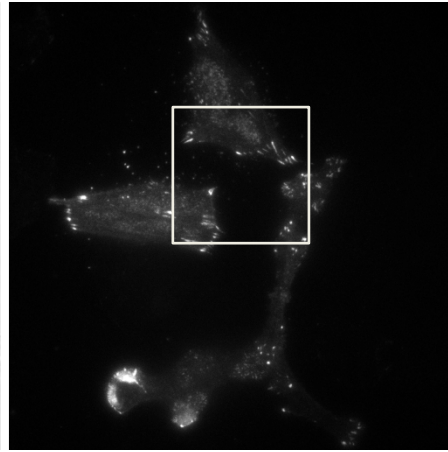

HER2-QD

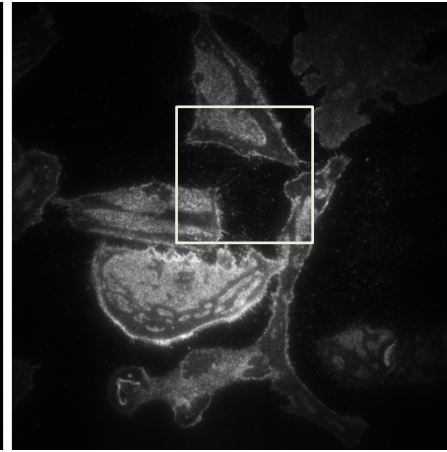

Merge

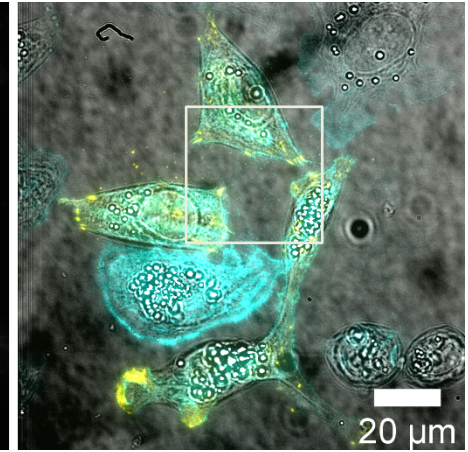

Supplement: S2 Fig — TIRF microscopy of SKBR3 cells transduced with talin-GFP on glass-bottom dishes analyzed with a 100x oil TIRF optimized objective. The intracellular domain of HER2 was labeled with a biotinylated ant-HER2 antibody coupled to strept-QD (HER2-QD). The same image as in Fig 3A is shown. The outline region indicates the magnified region shown Fig 3A. Shown are DIC, talin-GFP, HER2-QD fluorescence images and a merge image. Colors in merged image: yellow for GFP and cyan for HER2-QD. Scale bar: 20 μm. (PDF) [file pone.0234430.s002.pdf]

A

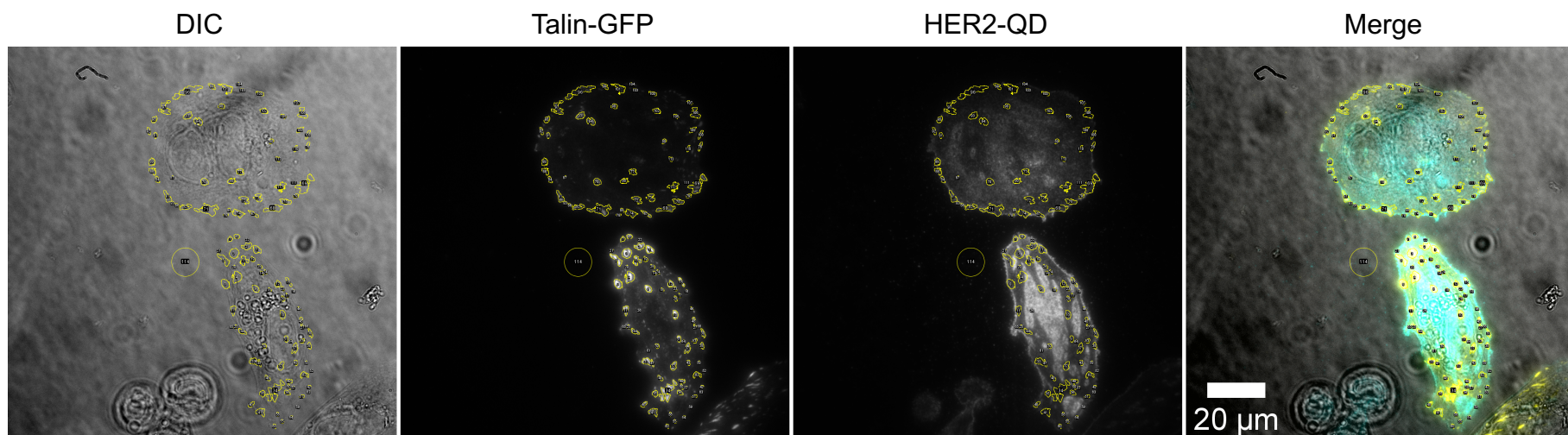

B

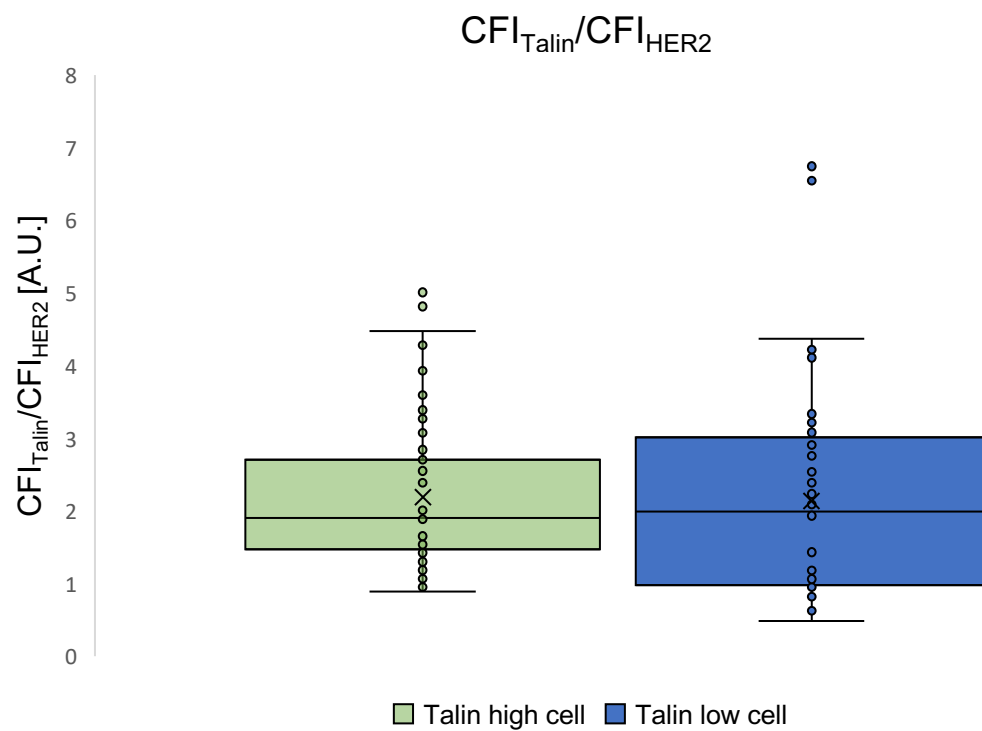

Supplement: S3 Fig — (A) DIC, talin-GFP, HER2-QD fluorescence images and merge image of SKBR3 cells acquired with TIRF (same image as in Fig 3B). Manually marked talin spots for fluorescence intensity analysis (B) are highlighted in all images (yellow). (B) Comparison of CFI ratios of talin to HER2 for talin high expressing cell (lower cell in S3A Fig) and low expressing cell (upper cell in Fig 3A). Similar ratios are seen for talin high (left) and talin low (right) expression. Each point represents one CFI ratio. n = 67 for the talin high expressing cell, n = 46 for the talin low expressing cell. Note that this analysis is part of the overall analysis shown in Fig 3D. Colors in merged image: yellow for GFP and cyan for HER2-QD. Scale bar: 20 μm. (PDF) [file pone.0234430.s003.pdf]
